# SegDesign: A modular framework for controllable protein segment engineering

**DOI:** 10.1002/pro.70542

**Published:** 2026-03-24

**Authors:** Chenjie Feng, Junbo Yin, Chao Zha, Mohammed Saif, Xiaopeng Xu, Xin Gao, Wenjia He

**Affiliations:** ^1^ Computer Science Program, Computer, Electrical and Mathematical Sciences and Engineering Division King Abdullah University of Science and Technology (KAUST) Thuwal Kingdom of Saudi Arabia; ^2^ College of Medical Information and Engineering Ningxia Medical University Yinchuan China; ^3^ Center of Excellence for Smart Health (KCSH) King Abdullah University of Science and Technology (KAUST) Thuwal Kingdom of Saudi Arabia; ^4^ Center of Excellence on Generative AI King Abdullah University of Science and Technology (KAUST) Thuwal Kingdom of Saudi Arabia

**Keywords:** local structural remodeling, protein backbone remodeling, protein design framework, protein engineering workflows, protein segment engineering, secondary‐structure control

## Abstract

Local protein structure engineering, such as targeted remodeling of loops or flexible regions, is critical for mechanistic studies and protein optimization but remains challenging to perform in a controllable and reproducible manner. Despite advances in protein foundation models and generative structure design, most existing methods emphasize global scaffold generation and offer limited support for precise, region‐specific intervention while preserving the overall fold. Here we present SegDesign, a modular framework for segment‐level protein engineering that integrates backbone reconstruction, sequence redesign, and multi‐stage structural evaluation into a unified workflow. SegDesign enables user‐defined secondary‐structure manipulation of selected regions and produces traceable, experimentally testable variant panels. Applied to *Gallus gallus* terminal deoxynucleotidyl transferase (*Gg*TdT), SegDesign converts an intrinsically flexible loop into either a stable α‐helix or β‐strand without disrupting the global fold, as supported by AlphaFold3 modeling. Applications to six additional enzymes further demonstrate the generality of the approach. SegDesign establishes an accessible paradigm for controllable local protein structure engineering in both mechanistic and applied settings. The standalone implementation of SegDesign is publicly available at https://github.com/mike114b/Segdesign.

## 1 INTRODUCTION

Local structural elements play a decisive role in shaping protein function (Heinemann et al. 2021; Nestl and Hauer 2014; Zeballos et al. 2025). Catalytic activity, thermal stability, substrate selectivity, allosteric communication, and inter‐domain coordination are frequently governed by the conformation, flexibility, and packing of short segments such as loops, α‐helices, β‐strands, linkers, and terminal regions (Nestl and Hauer 2014; Wu et al. 2021; Yu et al. 2017). As a result, many real‐world protein engineering tasks focus not on redesigning entire scaffolds but on precisely modifying a small set of residues, often on the order of a few to a few tens of amino acids, while leaving the global fold unchanged (Heinemann et al. 2021; Marek et al. 2020; Planas‐Iglesias et al. 2022; Yu et al. 2017). Common objectives include converting coiled loops into helices or strands to stabilize flexible regions, softening rigid elements to permit conformational transitions, extending secondary‐structure elements to reinforce folding stability, or tuning multi‐domain junctions to optimize communication (Karamitros et al. 2022; Marek et al. 2020; Nestl and Hauer 2014; Panigrahi et al. 2015; Song et al. 2015; Zinovjev et al. 2024). Beyond functional improvement, targeted segment remodeling can also facilitate intellectual‐property differentiation: modifying local structural motifs often reduces global sequence identity relative to existing patented enzymes, while still allowing activity and overall fold to be maintained.

Despite transformative advances in protein structure prediction and generative modeling, including diffusion‐based scaffold design (e.g., RFdiffusion; Watson et al. 2023), backbone‐conditioned sequence design (e.g., ProteinMPNN; Dauparas et al. 2022), and high‐fidelity structure prediction (e.g., ESMFold; Lin et al. 2023) and AlphaFold3 (Abramson et al. 2024), practical tools for fine‐grained, region‐specific structural remodeling remain limited. Most existing approaches are optimized for global or coarse‐grained design tasks, such as scaffold hallucination or motif‐level construction, and offer only limited control over user‐specified local regions. In parallel, sequence‐centric models generally lack explicit mechanisms to enforce desired secondary‐structure outcomes within predefined segments. As a result, even simple local engineering tasks, such as reshaping a single loop, typically require researchers to manually assemble heterogeneous components into bespoke pipelines that vary across laboratories, lack reproducibility, and remain inaccessible to users without substantial computational expertise (Liao et al. 2025; Weissenow and Rost 2025; Yang and Amini 2025; Zhu et al. 2025).

Such pipelines often involve ad hoc combinations of backbone sampling, manual filtering, and post hoc structural inspection, resulting in poor reproducibility, high dependence on user expertise, and limited accessibility to non‐specialists. Critically, there is currently no standardized or reproducible framework that enables users to explicitly prescribe secondary‐structure outcomes at user‐defined segments while systematically quantifying design success, failure modes, and global fold preservation across variants. This lack of principled, segment‐level control continues to hinder reproducibility, scalability, and cross‐study comparability in local protein structural remodeling.

This methodological gap is especially limiting for allostery, conformational dynamics, and enzyme design applications, where the behavior of a protein can hinge on the rigidity or flexibility of one or two short structural elements (Naganathan 2019; Reynolds et al. 2011; Vera‐Rodríguez et al. 2025; Zeballos et al. 2025). Increasing evidence suggests that local loops or β‐hairpins frequently act as dynamic hotspots or allosteric relays controlling long‐range communication (Mott and Owen 2018; Romero‐Tamayo et al. 2021; Wang et al. 2020). In industrial enzyme engineering, altering the stiffness or organization of such segments can markedly influence global thermostability, catalytic turnover, substrate channeling, or product release (Karamitros et al. 2022; Nestl and Hauer 2014; Song et al. 2015; Yu et al. 2017). However, experimentally probing these hypotheses requires the ability to harden a target loop (e.g., by introducing helix‐forming residues or β‐strands) or soften a rigid segment (e.g., by introducing coil elements), all while reliably preserving the overall scaffold (Karamitros et al. 2022; Marek et al. 2020; Sutthibutpong et al. 2018; Yu et al. 2017). Currently, there is no widely adopted computational framework that jointly generates, structurally validates, compares, and ranks localized structural variants in a unified, user‐friendly pipeline; instead, researchers typically combine multiple tools and ad hoc scripts for each new system.

To address these unmet needs, we developed SegDesign not as a standalone generative model, but as a methodological orchestration layer that formalizes segment‐level protein engineering into a reproducible, modular, and user‐controllable workflow. It integrates multiple foundation models and tools, including Hmmer (Mistry et al. 2013), DSSP (Hekkelman et al. 2025; Kabsch and Sander 1983; Touw et al. 2015), RFdiffusion (Watson et al. 2023), ProteinMPNN (Dauparas et al. 2022), and ESMFold (Lin et al. 2023), MMseqs2 (Mirdita et al. 2019; Steinegger and Söding 2017; Steinegger and Söding 2018), into a coherent workflow for targeted segment remodeling. Users can specify desired structural transitions, perform constrained sequence redesign, evaluate fold preservation alongside biophysical and developability metrics, and obtain ready‐to‐order variant panels with complete traceability. By transforming what was previously an ad hoc, script‐heavy process into a unified methodological framework, SegDesign lowers the barrier for mechanistic investigation, accelerates industrial enzyme optimization, and enables precise local engineering for both computational and experimental laboratories.

## 2 RESULTS

### 2.1 Overview of the SegDesign workflow

**FIGURE 1:**
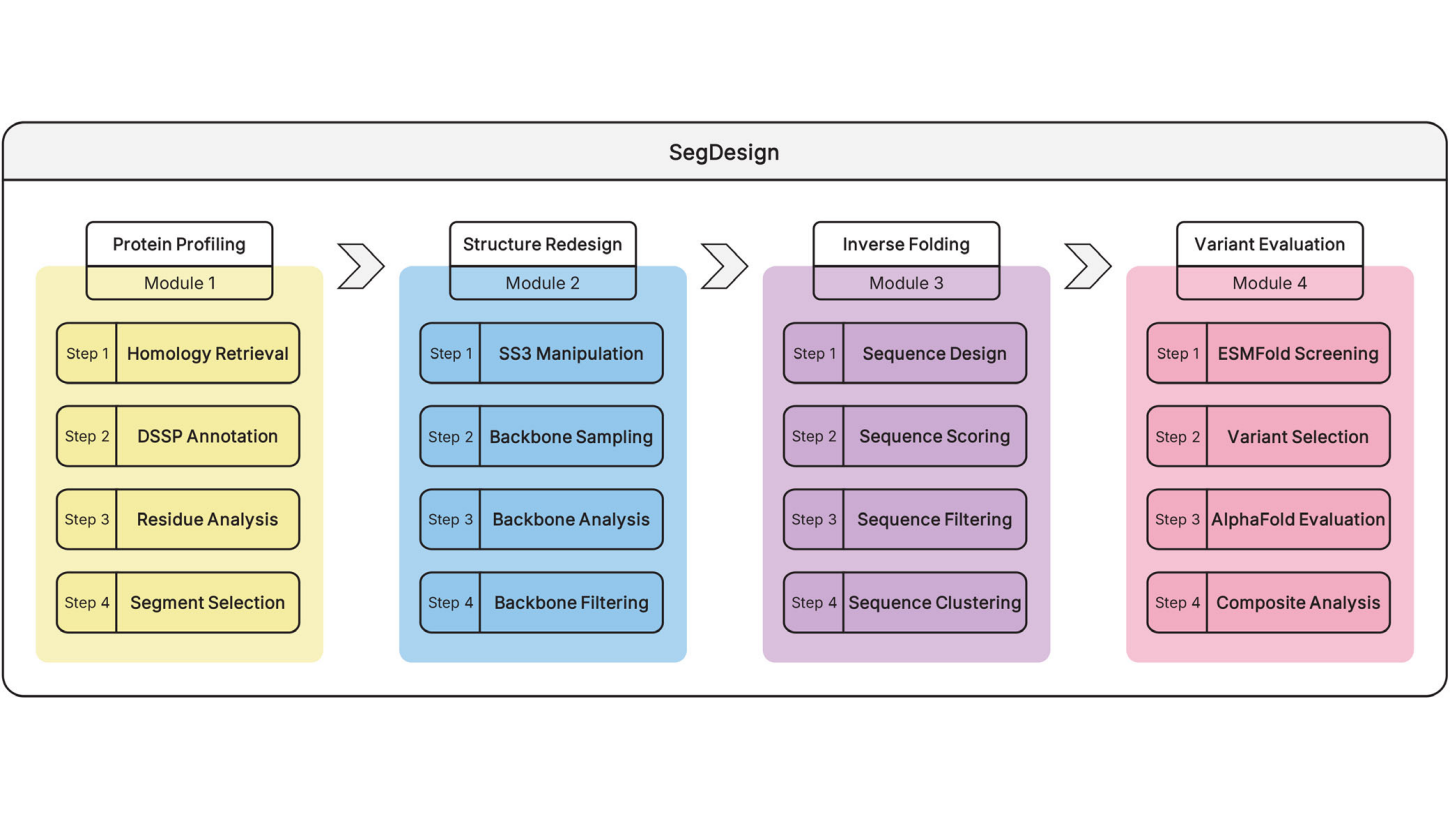
Overview of the SegDesign framework for modular protein segment design and evaluation. Schematic overview of the SegDesign pipeline, which integrates four sequential yet functionally decoupled modules to enable systematic and controllable local segment redesign. Module 1, Protein profiling, identifies designable regions through homology retrieval, DSSP‐based secondary‐structure annotation, residue‐level analysis, and target segment selection, providing structural and evolutionary context for downstream design. Module 2, Structure redesign, performs secondary‐structure–guided manipulation of the selected segments, followed by backbone sampling, structural analysis, and filtering to generate physically plausible backbone conformations. Module 3, Inverse folding, applies sequence design, scoring, filtering, and clustering to produce diverse sequence candidates compatible with the redesigned backbones. Module 4, Variant evaluation, conducts multi‐stage assessment including variant selection, rapid structural screening with ESMFold, high‐accuracy structure evaluation with AlphaFold3, and composite analysis to prioritize variants for downstream analysis or experimental validation. Together, SegDesign provides an interpretable, controllable, and scalable framework for targeted protein segment engineering beyond natural evolutionary constraints.

To enable precise and programmable remodeling of local protein structure, we developed SegDesign, an end‐to‐end, modular workflow for segment‐level protein engineering that integrates evolutionary profiling, backbone redesign, inverse folding, and multi‐stage structural evaluation (Figure 1). In contrast to existing protein design approaches that primarily operate at the global scaffold level or require extensive manual intervention, SegDesign adopts a segment‐centric design paradigm, allowing users to explicitly define both the target region and the intended secondary‐structure outcome while preserving the global fold.

SegDesign is built around a segment‐centric, objective‐driven paradigm, in which design intent is explicitly specified prior to generation rather than inferred post hoc. The workflow decomposes segment‐level protein engineering into four functionally decoupled modules, each addressing a distinct design question: (i) where the protein can be modified, (ii) what structural outcome is desired, (iii) which sequences are compatible with the redesigned backbone, and (iv) which variants are experimentally credible. Module 1 (Protein profiling) identifies designable segments by integrating homology retrieval, DSSP‐based secondary‐structure annotation, and conservation‐guided residue analysis, thereby delineating structurally flexible and evolutionarily permissive regions suitable for remodeling. Module 2 (Structure redesign) performs targeted backbone reconstruction of selected segments using RFdiffusion, followed by DSSP‐based validation and structural filtering to ensure that redesigned backbones adopt the user‐specified secondary‐structure class. Module 3 (Inverse folding) generates sequence variants compatible with the redesigned backbones via constrained inverse folding, coupled with systematic scoring, filtering, and clustering to produce diverse, high‐quality candidates. Finally, Module 4 (Variant evaluation) conducts multi‐level assessment, including rapid prescreening with ESMFold, optional high‐accuracy structure prediction with AlphaFold, and integrated comparative analysis to prioritize experimentally actionable designs.

Together, these modules establish a reproducible and extensible framework for local structural engineering, enabling fine‐grained secondary‐structure modulation while maintaining global fold integrity. By standardizing what is traditionally an ad hoc and labor‐intensive process, SegDesign provides a practical toolkit for function‐guided protein remodeling across diverse targets and applications, offering users both methodological rigor and flexible control over each stage of the design process.

### 2.2 Local secondary‐structure engineering of chicken TdT

Terminal deoxynucleotidyl transferase (TdT; EC 2.7.7.31) is a template‐independent DNA polymerase that catalyzes the addition of 2′‐deoxyribonucleoside 5′‐triphosphates (dNTPs) to the 3′ end of a DNA primer. In vertebrates, TdT plays a central role in V(D)J recombination by generating N‐nucleotide insertions, thereby contributing to antibody and T‐cell receptor diversity (Ashley et al. 2021; Mahmoud and Kearney 2010; Motea and Berdis 2010). Beyond its physiological function, TdT has emerged as a versatile biotechnological enzyme for applications including continuous DNA synthesis, single‐molecule sequencing, and molecular recording (Lu et al. 2022; Niu et al. 2025; Sachanka et al. 2025). However, its intrinsic structural flexibility, particularly within loop regions surrounding the catalytic pocket, poses challenges for thermostability, processivity, and precise control of polymerization, motivating the need for targeted local structural reinforcement without compromising catalytic function (Delarue et al. 2002; Motea and Berdis 2010; Mutt and Sowdhamini 2016).

**FIGURE 2:**
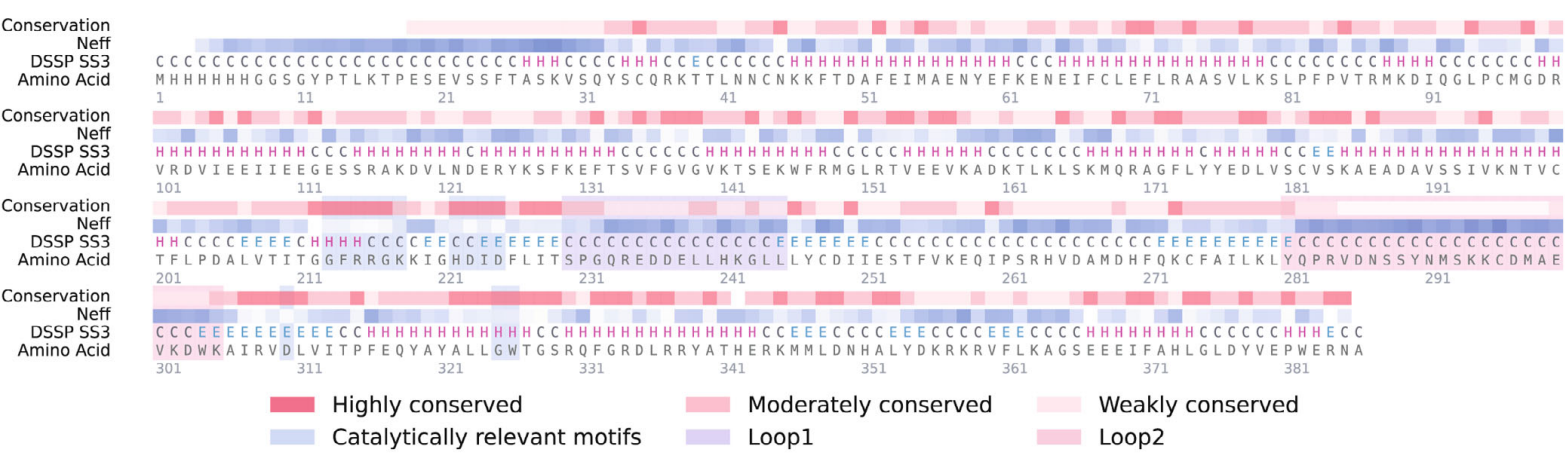
Structural and evolutionary profiling of GgTdT defines rational segment‐level design targets. Multi‐track representation of *Gg*TdT integrating evolutionary and structural features used to guide segment‐level design. From top to bottom, tracks depict the evolutionary profile, including per‐residue conservation level and the effective number of amino acids (Neff), followed by DSSP‐derived secondary‐structure annotation (SS3), and the primary sequence with residue indexing. Secondary‐structure elements are classified into α‐helices, β‐strands, and loops based on computational DSSP assignment. Evolutionary conservation is inferred from a multiple sequence alignment of homologous TdT proteins, enabling discrimination between evolutionarily constrained core regions and variable surface or loop segments. Catalytically relevant motifs and structurally flexible regions are annotated to provide functional context for design decisions. Two low‐conservation loop regions, denoted as Loop 1 and Loop 2, are highlighted as selected targets for subsequent secondary‐structure manipulation and redesign. Collectively, this integrated profiling establishes *Gg*TdT as a well‐characterized design target and provides a structural–evolutionary rationale for segment selection within the SegDesign framework.

We selected chicken TdT (Gallus gallus TdT, *Gg*TdT; UniProt P36195) as a representative target to demonstrate SegDesign's capability for controlled local secondary‐structure engineering. To simplify modeling and focus on the catalytic core, we removed the N‐terminal BRCT domain and introduced an N‐terminal His tag, consistent with prior studies showing that N‐terminal truncation improves expression and thermostability (Delarue et al. 2002; Li et al. 2024; Niu et al. 2025). Using the truncated catalytic domain as the design scaffold, we performed comprehensive structural and evolutionary profiling (Figure 2). DSSP‐based annotation and sequence conservation analysis revealed that the catalytic triad (Asp223, Asp225, and Asp310) and surrounding dNTP‐binding residues (positions 213–218, 222–225, and 325–326) are highly conserved and form a helix–strand composite architecture essential for enzymatic activity. In contrast, two surface‐exposed loop regions, Loop 1 (residues 230–245) and Loop 2 (residues 281–305), exhibit low sequence conservation and elevated structural flexibility, indicating that they are evolutionarily permissive and structurally amenable to targeted remodeling. Notably, in mouse TdT (UniProt P09838), the region corresponding to Loop 1 adopts a short α‐helical conformation, providing an evolutionary precedent for stabilizing this segment. Loop 2, comprising 25 residues, is highly solvent exposed and conformationally flexible, suggesting the potential to accommodate alternative secondary‐structure states.

Based on this profiling, we designed three distinct remodeling strategies to benchmark SegDesign’s ability to enforce user‐defined secondary‐structure outcomes in heterogeneous contexts: L1H, converting Loop 1 (230–245) from a predominantly coil conformation into a stable α‐helix; L2H, converting Loop 2 (281–305) into an α‐helical element to reduce local disorder; and L2S, converting Loop 2 into a β‐strand to bridge adjacent β‐strands within the surrounding sheet architecture. Together, these design targets provide a systematic testbed for evaluating SegDesign’s capacity to achieve precise and controllable local secondary‐structure remodeling while preserving the global fold.

Using SegDesign, we generated 100 candidate backbones for each design objective. DSSP‐based analysis revealed pronounced differences in backbone remodeling success across target regions. In GgTdT, loop‐to‐helix designs (L1H and L2H) achieved an average helical compliance of approximately 30%, whereas loop‐to‐strand designs (L2S) were substantially more challenging, with only ~12% average strand recovery. This pattern was consistently observed across identical loop regions, indicating an intrinsic asymmetry in local secondary‐structure designability rather than a limitation of the SegDesign pipeline itself.

The lower success rate of β‐strand induction can be rationalized by fundamental structural constraints associated with strand formation. In contrast to α‐helices, which are locally stabilized by intra‐chain hydrogen bonds and favorable backbone preferences, β‐strands rely on directional backbone hydrogen bonding with appropriate pairing partners and correct register alignment within a β‐sheet. As a result, successful strand formation depends not only on local backbone geometry but also on compatibility with the surrounding sheet topology. Moreover, β‐strand induction is highly sensitive to segment boundaries and local structural context: short or flexible loops often lack sufficient spatial constraints to support stable strand pairing, leading to designs that satisfy local geometric criteria yet fail to integrate coherently into a β‐sheet. Together, these observations highlight the intrinsically greater difficulty of β‐strand remodeling compared to α‐helix induction under otherwise identical design settings. By explicitly quantifying such differences, SegDesign provides a systematic framework for assessing and comparing segment‐level designability across distinct secondary‐structure classes.

**FIGURE 3:**
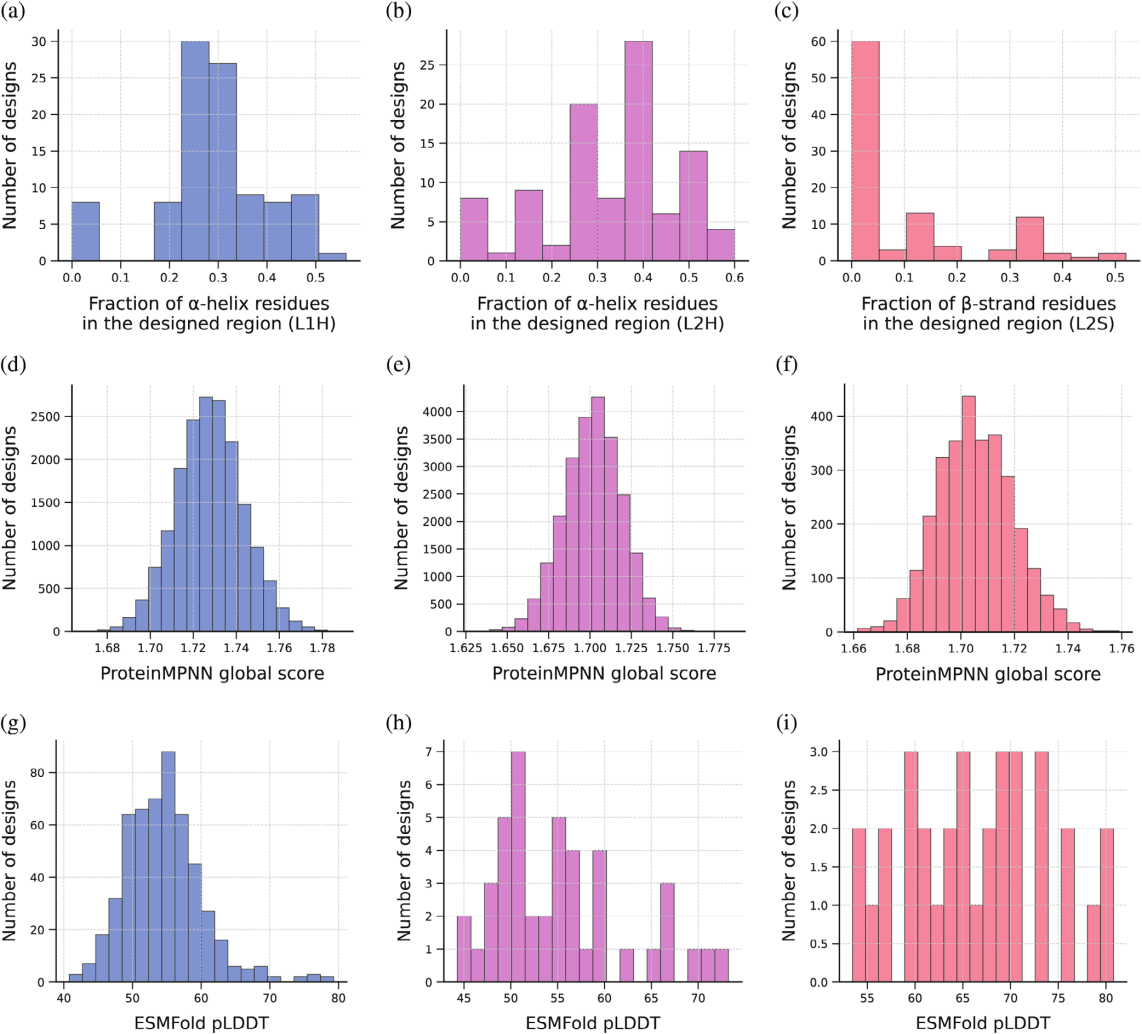
Secondary‐structure–guided redesign of unconserved loop regions in *Gg*TdT. Distribution of secondary‐structure realization, sequence quality, and structural confidence for designs generated under different segment‐level structural constraints. (a–c) Histograms showing the fraction of secondary‐structure residues within the redesigned regions for three design modes: Loop 1 redesigned as an α‐helix (L1H; a), Loop 2 redesigned as an α‐helix (L2H; b), and Loop 2 redesigned as a β‐strand (L2S; c). These distributions indicate that the imposed secondary‐structure constraints are effectively realized across the designed backbones while retaining structural diversity, with β‐strand enforcement exhibiting greater variability than α‐helical designs. (d–f) Corresponding distributions of ProteinMPNN global sequence scores for each design mode, with lower scores indicating higher sequence–structure compatibility. Comparable score distributions across L1H (d), L2H (e), and L2S (f) designs suggest that secondary‐structure manipulation of unconserved loop regions does not compromise overall sequence design quality. (g–i) Distributions of ESMFold‐predicted pLDDT scores for structurally successful variants, defined as designs achieving ≥40% target secondary‐structure coverage within the redesigned segment, for L1H (g), L2H (h), and L2S (i) design modes. The pLDDT distributions indicate that variants satisfying the secondary‐structure criteria also exhibit favorable local structural confidence, supporting the physical plausibility of the redesigned segments. Together, these analyses demonstrate that SegDesign enables controllable and segment‐specific secondary‐structure remodeling while maintaining sequence quality and structural confidence in the resulting variants.

Backbones exhibiting >40% compliance with the target secondary‐structure state were selected for sequence design using ProteinMPNN, with 1000 sequences sampled per backbone. The resulting ProteinMPNN global score distributions (Figure 3) were approximately Gaussian. Across different backbone templates, wild‐type sequences consistently scored near the median of the distribution, reflecting backbone‐dependent score normalization. Notably, a substantial fraction of designed sequences (>40%) achieved global scores superior to the wild type, suggesting that local secondary‐structure stabilization can be energetically favorable in specific structural contexts. To promote sequence diversity, the top 50% of sequences by ProteinMPNN global score were retained and clustered using MMseqs2, yielding hundreds of representative candidates for high‐throughput structural screening with ESMFold (Table S1, Supporting Information).

**FIGURE 4:**
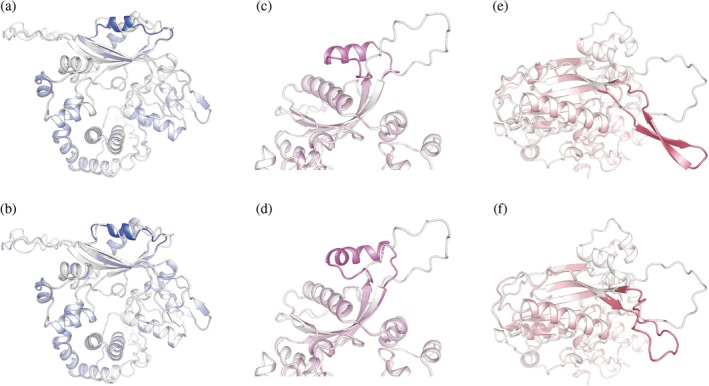
Structural realization of secondary‐structure–guided loop redesign in representative *Gg*TdT variants. AlphaFold3‐predicted structures of representative designed variants, structurally aligned to the wild‐type *Gg*TdT to enable direct comparison. (a, b) Representative variants generated under the Loop 1–to–α‐helix design mode (L1H, residues 230–245). (c, d) Representative variants generated under the Loop 2–to–α‐helix design mode (L2H, residues 281–305). (e, f) Representative variants generated under the Loop 2–to–β‐strand design mode (L2S, residues 281–305). In each panel, the redesigned segment is highlighted in a darker color, whereas the remainder of the protein is shown in a lighter, semi‐transparent representation. Structural alignment demonstrates that secondary‐structure remodeling is spatially confined to the targeted loop regions, while the global fold and overall domain architecture are preserved. These examples highlight the precision and controllability of segment‐level secondary‐structure redesign enabled by the SegDesign framework.

From the ESMFold‐filtered variant pool, representative designs were selected for high‐confidence structure prediction using AlphaFold3 and visualized in Figure 4, with corresponding quantitative details summarized in Table 1. Across all three design modes, structural alignment to the wild‐type GgTdT indicates that secondary‐structure remodeling is spatially confined to the targeted loop regions, while the global fold and overall domain architecture remain preserved. Detailed quantitative structural metrics are summarized in Table S2. Following a single global rigid‐body superposition, residues outside the redesigned segments (fixed regions) exhibit consistently low structural deviations, with Cα RMSD values ranging from approximately 2.7–3.6 Å and corresponding TM‐scores between 0.88 and 0.94 across all six representative variants. In contrast, the redesigned segments display substantially larger deviations, with Cα RMSD values spanning from ~3 Å to over 15 Å and markedly reduced TM‐scores (0.02–0.10), indicative of pronounced local structural remodeling. Importantly, this pattern is consistently observed across all three design strategies (L1H, L2H, and L2S), reinforcing that secondary‐structure manipulation remains spatially localized while preserving the overall scaffold architecture.

**TABLE 1:** Representative segment‐level secondary‐structure redesign variants of *Gg*TdT.

Strategy	Variant	Segment	Segment SS3 (before design)	Segment SS3 (after design)	Segment H%	Segment E%	Segment C%	Segment pLDDT
L1H	rfd_40_mpnn_399	230–245	CCCCCCCC CCCCCCCE	CCCCCCC**H** **HHHH**CCCC	**0.31**	0.00	0.69	70.5
	rfd_6_mpnn_862	230–245	CCCCCCCC CCCCCCCE	CCC**HHHHH** **HH**CCCCCC	**0.44**	0.00	0.56	73.1
L2H	rfd_54_mpnn_440	281–305	ECCCCCCCCCCC CCCCCCCCCCCEE	CC**HHH**CCC**HHHH** **HHHHHH**CC**HHH**CC	**0.64**	0.00	0.36	56.0
	rfd_69_mpnn_500	281–305	ECCCCCCCCCCC CCCCCCCCCCCEE	CCCCCC**HHHHHH** **HHHH**CCCCCCCCC	**0.40**	0.00	0.60	54.2
L2S	rfd_40_mpnn_381	281–305	ECCCCCCCCCCC CCCCCCCCCCCEE	C**E**CC**EEEEE**CCC CC**EEEEE**CCC**E**CC	0.00	**0.48**	0.52	58.9
	rfd_86_mpnn_508	281–305	ECCCCCCCCCCC CCCCCCCCCCCEE	C**EE**CCCCCCCCC CCCCCCCCC**EE**CC	0.00	**0.16**	0.84	50.2

*Note:* Boldface highlights the content of the intended secondary‐structure class for each redesign strategy, namely helix content (H%) for L2H variants and strand content (E%) for L2S variants.

For Loop 1 redesign (L1H; Figure 4a,b), variants rfd_40_mpnn_399 and rfd_6_mpnn_862 exhibit clear coil‐to‐helix transitions within residues 230–245. DSSP analysis (Table 1) indicates that the helical content in the redesigned segment increased from near‐zero in the wild type to 31–44%, accompanied by high segment‐level pLDDT values (>70), consistent with stable local helix formation without perturbing the surrounding scaffold.

For Loop 2 helix designs (L2H; Figure 4c,d), representative variants rfd_54_mpnn_440 and rfd_69_mpnn_500 show more extensive helix formation across residues 281–305, reaching up to 64% helical coverage (Table 1). Notably, these variants adopt distinct helical geometries and packing arrangements within the same loop region, illustrating that SegDesign does not converge to a single canonical solution but instead explores multiple structurally viable realizations of the target secondary structure.

In contrast, Loop 2 strand designs (L2S; Figure 4e,f) illustrate the increased challenge of enforcing β‐strand formation in flexible loops. Variants rfd_40_mpnn_381 and rfd_86_mpnn_508 display partial but well‐defined strand adoption, with β‐strand content reaching 16–48% (Table 1). Despite the lower absolute coverage relative to helix designs, AlphaFold3 models reveal coherent β‐strand segments that integrate into the local β‐sheet architecture while maintaining acceptable sequence–structure compatibility and segment‐level confidence.

Together, the structural visualizations in Figure 4 and the quantitative metrics in Table 1 demonstrate that SegDesign enables precise, programmable, and region‐specific secondary‐structure remodeling in *Gg*TdT. The framework supports both helix and strand induction, preserves global fold integrity, and generates multiple high‐confidence structural realizations for a given target region, providing a robust foundation for downstream mechanistic analysis and functional engineering.

### 2.3 Application across diverse enzymes demonstrates the generality of SegDesign

**FIGURE 5:**
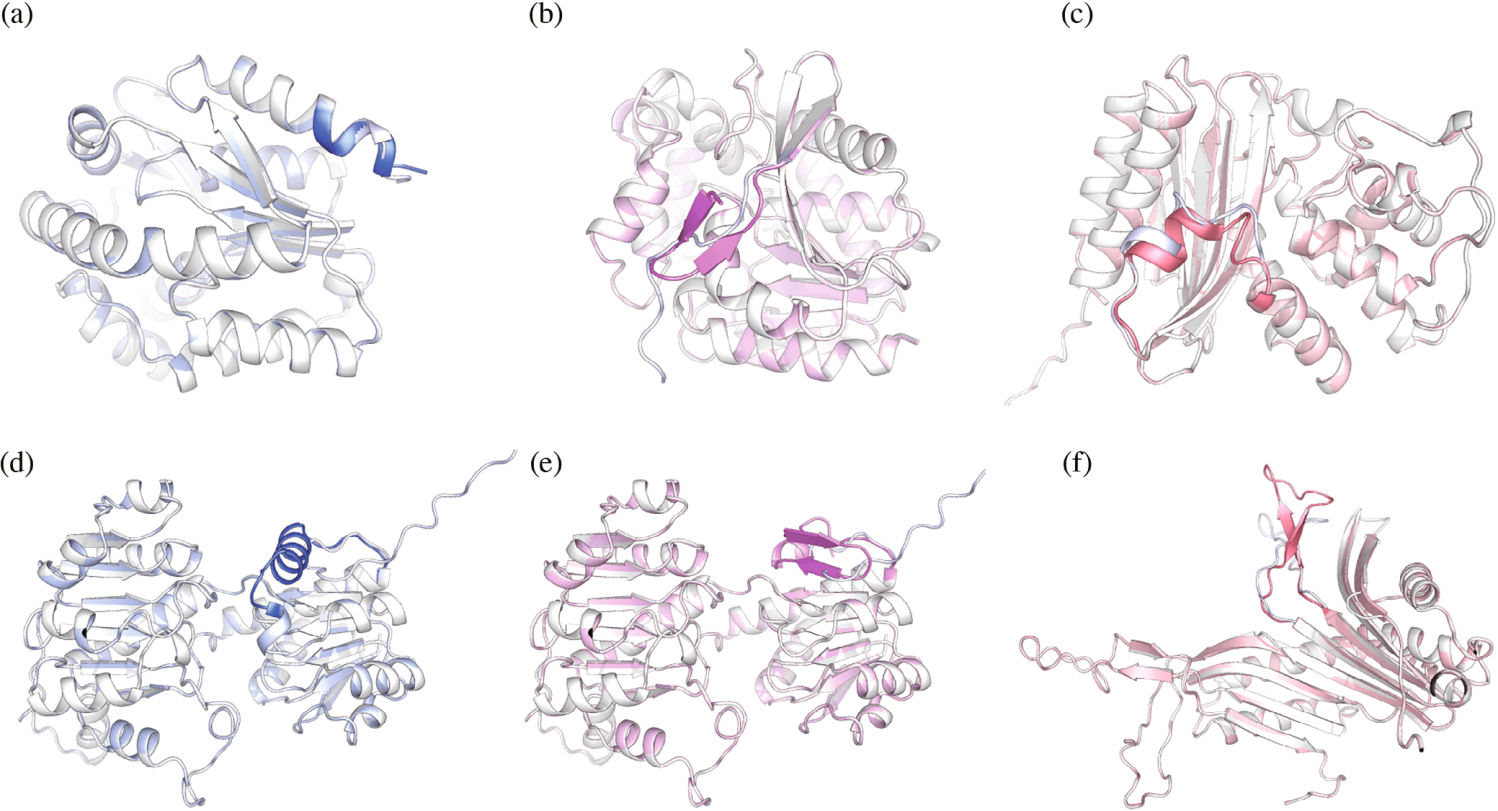
Generality of SegDesign for local secondary‐structure redesign across diverse enzymes. Representative AlphaFold3‐predicted structures of designed variants from multiple, structurally and functionally distinct enzymes, illustrating the general applicability of SegDesign beyond a single design target. Each designed structure is structurally aligned to its corresponding wild‐type enzyme to enable direct comparison. (a) Adenylate kinase, in which a C‐terminal segment (residues 211–217) was redesigned to extend and stabilize an α‐helical element. (b) Haloalkane dehalogenase, where the N‐terminal segment (residues 1–12) was remodeled into a β‐strand to stabilize an otherwise flexible terminus. (c) TEM β‐lactamase, showing helix extension within an internal segment (residues 211–225). (d, e) *Klebsiella pneumoniae* D‐lactate dehydrogenase (LDHD), in which the same C‐terminal segment (residues 129–145) was independently redesigned into an α‐helix (d) or a β‐strand (e) to benchmark distinct secondary‐structure outcomes in an intrinsically flexible region. (f) *Aspergillus flavus* urate oxidase (UOX), where an internal segment (residues 104–128) was remodeled to promote β‐strand formation. In each panel, the redesigned segment is highlighted in a darker color, whereas the remainder of the protein is shown in a lighter, semi‐transparent representation. Structural alignment demonstrates that secondary‐structure remodeling is spatially confined to the targeted loop regions, while the global fold and overall domain architecture are preserved.

Beyond TdT, we applied SegDesign to a panel of enzymes specifically chosen to stress‐test the framework across diverse folds, segment locations, and structural contexts, rather than to showcase isolated successes (Table 2). This panel, including adenylate kinase, haloalkane dehalogenase, TEM β‐lactamase, *Klebsiella pneumoniae* D‐lactate dehydrogenase (LDHD), and *Aspergillus flavus* urate oxidase (UOX), spans multiple enzyme classes and catalytic mechanisms and is widely used in energy metabolism, biocatalysis, antibiotic resistance studies, industrial biotransformation, and biosensing applications. For each enzyme, we targeted structurally flexible loop regions, preferentially located at the N‐ or C‐termini, which are generally more permissive and evolutionarily unconstrained, thereby minimizing confounding effects when assessing the controllability of local secondary‐structure remodeling. Representative redesigned variants are shown in Figure 5, with detailed information provided in Table S3.

**TABLE 2:** Diverse enzymes and segment‐level design targets used in this study.

Enzyme	Database	Accession	Designed segment	Target secondary structure
Adenylate kinase	UniProt	P16304	211–217	α‐helix
Haloalkane dehalogenase	UniProt	P59336	1–12	β‐strand
Beta‐lactamase TEM	UniProt	P62593	211–225	α‐helix
Klebsiella pneumoniae D‐lactate dehydrogenase (LDHD)	NCBI	WP_011673315.1	129–145	α‐helix
Klebsiella pneumoniae D‐lactate dehydrogenase (LDHD)	NCBI	WP_011673315.1	129–145	β‐strand
Aspergillus flavus urate oxidase (UOX)	UniProt	Q00511	104–128	β‐strand

For **adenylate kinase** (Figure 5a), the C‐terminal segment extends from a helical region and terminates in three residues annotated as coil, suggesting incomplete structural stabilization. We therefore targeted this terminal segment for helix extension. SegDesign successfully converted four of the seven terminal residues into a helical conformation, specifically adopting a 3_10_‐helix geometry (DSSP SS8 = “G”), thereby stabilizing the local C‐terminal architecture.

For **haloalkane dehalogenase** (Figure 5b), the N‐terminal 12 residues are annotated as coil and lack defined secondary structure. We aimed to stabilize this region by enforcing β‐strand formation. SegDesign folded the N‐terminal segment into a short antiparallel β‐strand comprising three residues, demonstrating precise control over secondary‐structure induction at protein termini.

To further assess remodeling of intrinsically disordered regions, we examined ***Klebsiella pneumoniae* D‐lactate dehydrogenase** (Figure 5d,e), whose C‐terminal 26 residues exhibit pronounced disorder. We independently targeted this segment for helix‐ and strand‐based remodeling. SegDesign achieved a 62% helix compliance in the helix‐enforced design (Figure 5d) and a 35% strand compliance in the strand‐enforced design (Figure 5e), again highlighting the greater difficulty of stabilizing β‐strands relative to α‐helices in flexible regions.

For **TEM β‐lactamase** (Figure 5c), we targeted an internal helical segment (residues 211–225) rather than a terminal region, aiming to extend an existing helix within the protein core. SegDesign increased the helical coverage of this segment from **27% to 60%**, demonstrating its applicability to non‐terminal and structurally embedded regions.

Finally, in **A. flavus urate oxidase** (Figure 5f), we targeted an internal segment (residues 104–128) for β‐strand reinforcement. SegDesign remodeled this region into an antiparallel β‐strand spanning six residues, achieving approximately **60% β‐strand coverage**, further supporting its ability to induce strand formation in diverse structural contexts.

Together, these case studies demonstrate that SegDesign generalizes across enzymes with distinct folds, functional roles, and design objectives, enabling controllable secondary‐structure remodeling at both terminal and internal regions. The consistent observation that β‐strand enforcement remains more challenging than α‐helical stabilization underscores a shared biophysical constraint across systems, while highlighting SegDesign's capacity to systematically probe and manipulate such local structural features in a programmable manner.

## 3 DISCUSSION AND CONCLUSIONS

### 3.1 Positioning of SegDesign within local protein engineering

Local structural engineering represents a critical yet historically underdeveloped dimension of protein design. Although recent generative models have enabled impressive advances in de novo fold hallucination and global topology refinement, they offer limited control over precise, region‐specific remodeling. In many practical protein engineering scenarios, however, the overall scaffold must remain intact, and only a small number of residues or segments require targeted intervention. SegDesign addresses this unmet need by formalizing segment‐level protein engineering into a standardized, reproducible, and accessible workflow.

A central conceptual advance of SegDesign is the reframing of local structural remodeling from a largely trial‐and‐error procedure into a hypothesis‐driven design operation. By explicitly specifying structural intent at the segment level and systematically evaluating design outcomes, SegDesign enables controlled exploration of local structural space while preserving global fold integrity. Across multiple case studies, the framework consistently achieves localized secondary‐structure transitions, including loop‐to‐helix and loop‐to‐strand conversion as well as secondary‐structure extension, supported by high‐confidence AlphaFold3 predictions.

### 3.2 Added value beyond ad hoc combinations of existing tools

SegDesign differs from existing methods such as inverse‐folding or loop‐modeling practices in the degree of user control and standardization it provides. Inverse‐folding models (e.g., ProteinMPNN) primarily optimize sequences conditioned on a fixed backbone, and thus cannot directly enforce a prescribed secondary‐structure transition when the backbone itself must be remodeled. Fragment‐based or loop modeling approaches can generate local conformations, but typically require manual choices of fragments, scoring terms, and iterative human inspection, making outcomes difficult to reproduce across laboratories. By contrast, SegDesign explicitly defines a segment‐level design objective (target region + SS3 outcome), couples backbone regeneration with objective‐driven filtering, and reports success/failure statistics across variants, thereby enabling programmable secondary‐structure editing under global fold constraints.

In principle, local secondary‐structure manipulation can be attempted by manually combining backbone generators and inverse‐folding models. However, such ad hoc workflows typically lack explicit objective specification, standardized filtering criteria, and reproducible reporting of intermediate design decisions. SegDesign addresses these limitations by embedding segment‐level design goals within a unified, multi‐stage workflow.

By explicitly defining target regions and desired secondary‐structure outcomes, applying consistent filtering and ranking schemes, and recording intermediate results throughout the design process, SegDesign enables systematic comparison of design outcomes and reliable assessment of success and failure modes across different structural objectives. In this sense, SegDesign functions as an orchestration layer that bridges powerful but monolithic foundation models and real‐world protein engineering workflows. While models such as RFdiffusion, ProteinMPNN, and ESMFold provide strong generative and predictive capabilities, their effective use for fine‐grained geometric control often requires extensive manual integration. SegDesign abstracts this complexity into a coherent pipeline, enabling programmable local remodeling without ad hoc scripting and improving both reproducibility and accessibility.

### 3.3 Practical utility and user accessibility

SegDesign is designed to serve a broad spectrum of users, ranging from experimental researchers seeking a deployable solution for routine segment‐level protein engineering to advanced users interested in customizing individual design stages. For users with limited programming experience, the framework can be executed end‐to‐end using predefined configurations and default scripts, enabling straightforward application without extensive code modification. More advanced users may selectively run, modify, or replace individual modules to accommodate customized objectives or alternative design strategies.

Importantly, SegDesign does not require in‐depth expertise in machine learning. Model‐specific details are abstracted through modular implementations and default settings, while basic familiarity with structural biology concepts and command‐line execution is sufficient for effective use. Within this design space, SegDesign supports a range of common protein engineering tasks that require localized structural intervention while preserving the global fold, including loop stabilization, targeted secondary‐structure conversion, and local rigidity tuning for mechanistic or functional studies.

### 3.4 From structural design to experimental validation

By integrating secondary‐structure objectives, designability constraints, and structural confidence metrics into automated filtering and ranking schemes, SegDesign facilitates rapid design–test cycles. The resulting variant panels are directly suitable for experimental follow‐up, accelerating the transition from computational hypotheses to empirical validation. Moreover, the modular architecture of SegDesign ensures extensibility: emerging models for structure generation, sequence design, or foldability prediction can be readily incorporated, allowing the framework to evolve alongside advances in protein AI.

Beyond individual design tasks, SegDesign also enables systematic quantification of segment‐level designability across different secondary‐structure classes and local contexts. Such analyses provide insight into intrinsic asymmetries between structural motifs and offer a principled basis for comparing local remodeling difficulty across proteins and regions.

### 3.5 Limitations and future directions

Several limitations merit consideration. SegDesign currently relies on static structure predictors, and its performance may be reduced for intrinsically disordered or highly dynamic regions where single‐structure representations are insufficient. In addition, the framework prioritizes structural endpoints and does not explicitly model conformational dynamics, kinetic effects, or functional transition pathways. Achieving high compliance with prescribed secondary‐structure states, particularly for short or highly flexible loops, may therefore require iterative refinement across multiple design rounds.

At the same time, these limitations highlight clear avenues for extension. By enabling well‐controlled, localized structural perturbations at predefined segments, SegDesign provides an ideal substrate for future integration with dynamics‐aware scoring functions, molecular simulations, and experimental feedback loops. Incorporating automated multi‐round optimization, ensemble‐based evaluation, or kinetic‐aware metrics could further improve robustness and success rates, positioning SegDesign as a foundational orchestration layer for next‐generation segment‐level protein design methodologies.

### 3.6 Conclusions

Overall, SegDesign advances protein engineering toward a more modular, interpretable, and user‐centric paradigm. By enabling controllable local structural manipulation at scale, it facilitates systematic interrogation of structure–function relationships and provides a low‐risk strategy for protein and enzyme optimization in both academic and industrial contexts. Beyond its immediate applications, SegDesign also offers a principled platform for generating high‐quality datasets that can inform future models explicitly designed to learn the rules governing local protein structure remodeling.

## 4 MATERIALS AND METHODS

SegDesign is a modular and fully reproducible workflow for targeted remodeling of local protein segments. The pipeline accepts a protein structure (PDB or mmCIF format) as input, together with optional user‐defined design regions (e.g., residues 376–431) and desired secondary‐structure objectives (e.g., α‐helix or β‐strand). SegDesign is organized into four sequential yet functionally decoupled modules (M1–M4), corresponding to protein profiling, structure redesign, inverse folding, and variant evaluation (Figure 1). Each module produces intermediate outputs, including structural snapshots, logs, and quality‐control summaries, enabling full traceability and reproducibility. Final outputs comprise curated sequence variants, predicted structures, and multi‐metric assessments suitable for downstream experimental validation.

### 4.1 Module 1: Protein profiling

#### 4.1.1 Homology retrieval

To characterize evolutionary constraints and identify regions amenable to redesign, homologous sequences were retrieved using JackHMMER with E‐value thresholds ranging from 1 × 10^−3^ to 1 × 10^−5^ and maximum hit counts of 5000–50,000, depending on computational resources. Redundancy was reduced by clustering sequences at 80% identity using *hhfilter*, stabilizing conservation estimates.

#### 4.1.2 Secondary‐structure annotation

Secondary‐structure states were assigned using DSSP for either experimentally determined structures or predicted models, yielding both SS3 and SS8 annotations. These assignments were used to characterize local structural environments and to identify helices, strands, and loop regions as potential redesign targets.

#### 4.1.3 Residue‐level analysis and segment selection

Designability was assessed by integrating evolutionary conservation, structural context, and biochemical considerations. Per‐residue conservation was quantified using the effective number of amino acids (Neff). Structural features such as DSSP state were incorporated, with flexible coil regions preferentially highlighted. Regions combining low evolutionary constraint with non‐essential structural roles were selected as designable segments.

### 4.2 Module 2: Structure redesign

#### 4.2.1 Secondary‐structure manipulation

For each selected segment, the corresponding backbone region was released for redesign, while the remainder of the structure was treated as a fixed scaffold. Positional constraints were applied to non‐designed residues to preserve the global topology and user‐specified secondary‐structure objectives that defined the intended local transformation. In SegDesign, secondary‐structure targets are implemented primarily as an objective‐driven, post‐generation (soft) constraint, with optional guidance during backbone generation. RFdiffusion is used to generate locally inpainted backbone conformations within the designated segment, where the target secondary structure can be provided as a soft conditioning signal rather than a hard constraint. As a result, generated backbones are not guaranteed to satisfy the intended secondary‐structure outcome. The resulting structures are therefore subsequently evaluated using DSSP to quantify SS3 compliance. Candidate backbones are then filtered and ranked based on the fraction of residues matching the target SS3 state, ensuring that only designs meeting the expected structural objective are retained.

#### 4.2.2 Backbone sampling

Local backbone regeneration was performed using RFdiffusion in inpainting mode, enabling loop‐to‐helix or loop‐to‐strand conversion, extension of existing secondary‐structure elements, and stabilization of flexible motifs. Sampling was strictly confined to the designated segment to preserve the global scaffold. The number of diffusion steps was set to 50, which empirically improved both the success rate and structural diversity of secondary‐structure remodeling by allowing sufficient exploration of local conformational space rather than converging prematurely to minimally perturbed backbone configurations.

#### 4.2.3 Structural assessment and filtering

All sampled backbones were reannotated using DSSP to evaluate compliance with the target secondary structure. Candidates were ranked by the fraction of residues adopting the intended SS3 state, and models deviating substantially from the design objective were removed. Remaining structures were further filtered for geometric continuity, steric plausibility, and boundary compatibility, yielding a refined set of backbone templates for sequence design.

### 4.3 Module 3: Inverse folding

#### 4.3.1 Sequence design and scoring

For each validated backbone, multiple candidate sequences were generated using inverse‐folding models, with ProteinMPNN as the primary engine. Sampling temperatures were adjusted to balance diversity and sequence–structure compatibility. Designs were evaluated using model‐derived global scores and annotated with backbone provenance and sampling parameters to ensure systematic tracking and downstream comparability.

#### 4.3.2 Filtering and clustering

Sequences failing to meet minimal structural or score‐based criteria were discarded. The remaining designs were clustered using MMseqs2 to reduce redundancy, and representative sequences from each cluster were retained. The minimum sequence identity threshold (min_seq_id) was adaptively adjusted across different design segments and secondary‐structure objectives to maintain the final set of representative variants at a scale of several hundred, balancing design quality, computational efficiency, and sequence diversity for downstream structural prescreening.

### 4.4 Module 4: Variant evaluation

#### 4.4.1 Rapid structural screening

Candidate sequences were folded using ESMFold for high‐throughput structural prescreening. Designs were filtered based on both local and global confidence metrics, including pLDDT values within the redesigned segment, the coverage of the target secondary‐structure state in the design region, and the global mean pLDDT, ensuring that local remodeling was achieved without compromising overall structural quality.

#### 4.4.2 High‐accuracy structure evaluation and composite analysis

Selected variants were optionally evaluated using AlphaFold3 to obtain higher‐confidence structure predictions. Structural confidence, backbone geometry, and consistency with the intended secondary‐structure remodeling were assessed. PyMOL‐based visualization and local conformational inspection were performed for preliminary manual quality assessment. In parallel, a composite analysis integrating ProteinMPNN global scores, ESMFold‐derived pLDDT, DSSP‐assigned secondary‐structure states, and structural deviations in non‐designed regions was conducted to prioritize high‐quality, well‐behaved variants.

### 4.5 Reproducibility and user guidelines

To support reproducibility, all parameters and filtering criteria used in SegDesign are summarized in Table S4. Detailed user guidelines, including recommended parameter ranges and computational considerations, are provided in the Supporting Information.

## AUTHOR CONTRIBUTIONS

**Chenjie Feng:** Methodology; validation; software; writing – review and editing; funding acquisition; project administration; resources; supervision. **Junbo Yin:** Validation; methodology; writing – review and editing. **Chao Zha:** Writing – review and editing; methodology; software. **Mohammed Saif:** Methodology; writing – review and editing; investigation. **Xiaopeng Xu:** Methodology; writing – review and editing; visualization. **Xin Gao:** Funding acquisition; writing – review and editing; supervision; resources; project administration. **Wenjia He:** Conceptualization; investigation; methodology; validation; visualization; writing – original draft; writing – review and editing; supervision; formal analysis; project administration.

## CONFLICT OF INTEREST STATEMENT

The authors declare no conflicts of interest.

## Supporting information


**Table S1.** Detailed information for GgTdT design.
**Table S2.** Alignment metrics for fixed regions and redesigned segments in GgTdT variants.
**Table S3.** Representative segment‐level secondary‐structure redesign variants of various enzymes.
**Table S4.** Parameter summary for SegDesign workflow.

## Data Availability

Standalone‐version of SegDesign is available at https://github.com/mike114b/Segdesign. The data used in this research can also be downloaded from GitHub.

## References

[pro70542-bib-0001] Abramson J , Adler J , Dunger J , Evans R , Green T , Pritzel A , et al. Accurate structure prediction of biomolecular interactions with AlphaFold 3. Nature. 2024;630:493–500. 38718835 10.1038/s41586-024-07487-w PMC11168924

[pro70542-bib-0002] Ashley J , Schaap‐Johansen AL , Mohammadniaei M , Naseri M , Marcatili P , Prado M , et al. Terminal deoxynucleotidyl transferase‐mediated formation of protein binding polynucleotides. Nucleic Acids Res. 2021;49:1065–1074. 33398328 10.1093/nar/gkaa1263 PMC7826267

[pro70542-bib-0004] Dauparas J , Anishchenko I , Bennett N , Bai H , Ragotte RJ , Milles LF , et al. Robust deep learning–based protein sequence design using ProteinMPNN. Science. 2022;378:49–56. 36108050 10.1126/science.add2187 PMC9997061

[pro70542-bib-0005] Delarue M , Boulé JB , Lescar J , Expert‐Bezançon N , Jourdan N , Sukumar N , et al. Crystal structures of a template‐independent DNA polymerase: murine terminal deoxynucleotidyltransferase. EMBO J. 2002;21:427–439. 11823435 10.1093/emboj/21.3.427 PMC125842

[pro70542-bib-0006] Heinemann PM , Armbruster D , Hauer B . Active‐site loop variations adjust activity and selectivity of the cumene dioxygenase. Nat Commun. 2021;12:1095. 33597523 10.1038/s41467-021-21328-8 PMC7889853

[pro70542-bib-0007] Hekkelman ML , Salmoral DÁ , Perrakis A , Joosten RP . DSSP 4: FAIR annotation of protein secondary structure. Protein Sci. 2025;34:e70208. 40671631 10.1002/pro.70208 PMC12268231

[pro70542-bib-0008] Kabsch W , Sander C . Dictionary of protein secondary structure: pattern recognition of hydrogen‐bonded and geometrical features. Biopolymers. 1983;22:2577–2637. 6667333 10.1002/bip.360221211

[pro70542-bib-0009] Karamitros CS , Murray K , Winemiller B , Lamb C , Stone EM , D'Arcy S , et al. Leveraging intrinsic flexibility to engineer enhanced enzyme catalytic activity. Proc Natl Acad Sci. 2022;119:e2118979119. 35658075 10.1073/pnas.2118979119 PMC9191678

[pro70542-bib-0010] Li AN , Shi K , Zeng BB , Xu JH , Yu HL . Enhancing the expression of terminal deoxynucleotidyl transferases by N‐terminal truncation. Biotechnol J. 2024;19:2400226. 10.1002/biot.202400226 39295567

[pro70542-bib-0011] Liao S , Xu G , Jin L , Ma J . De novo design of large polypeptides using a lightweight diffusion model integrating LSTM and attention mechanism under per‐residue secondary structure constraints. Molecules. 2025;30:1116. 40076339 10.3390/molecules30051116 PMC11902264

[pro70542-bib-0012] Lin Z , Akin H , Rao R , Hie B , Zhu Z , Lu W , et al. Evolutionary‐scale prediction of atomic‐level protein structure with a language model. Science. 2023;379:1123–1130. 36927031 10.1126/science.ade2574

[pro70542-bib-0013] Lu X , Li J , Li C , Lou Q , Peng K , Cai B , et al. Enzymatic DNA synthesis by engineering terminal deoxynucleotidyl transferase. ACS Catal. 2022;12:2988–2997.

[pro70542-bib-0014] Mahmoud TI , Kearney JF . Terminal deoxynucleotidyl transferase is required for an optimal response to the polysaccharide α‐1, 3 dextran. J Immunol. 2010;184:851–858. 20018621 10.4049/jimmunol.0902791 PMC4710363

[pro70542-bib-0015] Marek M , Chaloupkova R , Prudnikova T , Sato Y , Rezacova P , Nagata Y , et al. Structural and catalytic effects of surface loop‐helix transplantation within haloalkane dehalogenase family. Comput Struct Biotechnol J. 2020;18:1352–1362. 32612758 10.1016/j.csbj.2020.05.019 PMC7306515

[pro70542-bib-0016] Mirdita M , Steinegger M , Söding J . MMseqs2 desktop and local web server app for fast, interactive sequence searches. Bioinformatics. 2019;35:2856–2858. 30615063 10.1093/bioinformatics/bty1057 PMC6691333

[pro70542-bib-0017] Mistry J , Finn RD , Eddy SR , Bateman A , Punta M . Challenges in homology search: HMMER3 and convergent evolution of coiled‐coil regions. Nucleic Acids Res. 2013;41:e121. 23598997 10.1093/nar/gkt263 PMC3695513

[pro70542-bib-0018] Motea EA , Berdis AJ . Terminal deoxynucleotidyl transferase: the story of a misguided DNA polymerase. Biochim Biophys Acta. 2010;1804:1151–1166. 19596089 10.1016/j.bbapap.2009.06.030 PMC2846215

[pro70542-bib-0019] Mott HR , Owen D . Allostery and dynamics in small G proteins. Biochem Soc Trans. 2018;46:1333–1343. 30301845 10.1042/BST20170569

[pro70542-bib-0020] Mutt E , Sowdhamini R . Molecular dynamics simulations and structural analysis to decipher functional impact of a twenty residue insert in the ternary complex of Mus musculus TdT isoform. PLoS One. 2016;11:e0157286. 27311013 10.1371/journal.pone.0157286 PMC4911049

[pro70542-bib-0021] Naganathan AN . Modulation of allosteric coupling by mutations: from protein dynamics and packing to altered native ensembles and function. Curr Opin Struct Biol. 2019;54:1–9. 30268910 10.1016/j.sbi.2018.09.004 PMC6420056

[pro70542-bib-0022] Nestl BM , Hauer B . Engineering of flexible loops in enzymes. ACS Catl. 2014;4:3201–3211.

[pro70542-bib-0023] Niu Y , Chen B , Zhang H , Zheng W , Wu J , Yang L , et al. Computational design of a thermostable and highly active terminal deoxynucleotidyl transferase for synthesis of long de novo DNA molecules. ACS Catal. 2025;15:7201–7216.

[pro70542-bib-0024] Panigrahi P , Sule M , Ghanate A , Ramasamy S , Suresh C . Engineering proteins for thermostability with iRDP web server. PLoS One. 2015;10:e0139486. 26436543 10.1371/journal.pone.0139486 PMC4593602

[pro70542-bib-0025] Planas‐Iglesias J , Opaleny F , Ulbrich P , Stourac J , Sanusi Z , Pinto GP , et al. LoopGrafter: a web tool for transplanting dynamical loops for protein engineering. Nucleic Acids Res. 2022;50:W465–W473. 35438789 10.1093/nar/gkac249 PMC9252738

[pro70542-bib-0026] Reynolds KA , McLaughlin RN , Ranganathan R . Hot spots for allosteric regulation on protein surfaces. Cell. 2011;147:1564–1575. 22196731 10.1016/j.cell.2011.10.049 PMC3414429

[pro70542-bib-0027] Romero‐Tamayo S , Laplaza R , Velazquez‐Campoy A , Villanueva R , Medina M , Ferreira P . W196 and the β‐hairpin motif modulate the redox switch of conformation and the biomolecular interaction network of the apoptosis‐inducing factor. Oxid Med Cell Longev. 2021;2021:6673661. 33510840 10.1155/2021/6673661 PMC7822688

[pro70542-bib-0028] Sachanka AB , Douhaya SS , Shchur VV , Yantsevich AV . Enhancement of the nucleotide incorporation activity of the terminal deoxynucleotidyl transferase by N‐terminal truncation and DNA‐binding protein modulation. Biochim Biophys Acta. 2025;1874:141114. 10.1016/j.bbapap.2025.141114 41232740

[pro70542-bib-0029] Song L , Tsang A , Sylvestre M . Engineering a thermostable fungal GH10 xylanase, importance of N‐terminal amino acids. Biotechnol Bioeng. 2015;112:1081–1091. 25640404 10.1002/bit.25533

[pro70542-bib-0030] Steinegger M , Söding J . MMseqs2 enables sensitive protein sequence searching for the analysis of massive data sets. Nat Biotechnol. 2017;35:1026–1028. 29035372 10.1038/nbt.3988

[pro70542-bib-0031] Steinegger M , Söding J . Clustering huge protein sequence sets in linear time. Nat Commun. 2018;9:2542. 29959318 10.1038/s41467-018-04964-5 PMC6026198

[pro70542-bib-0032] Sutthibutpong T , Rattanarojpong T , Khunrae P . Effects of helix and fingertip mutations on the thermostability of xyn11A investigated by molecular dynamics simulations and enzyme activity assays. J Biomol Struct Dyn. 2018;36:3978–3992. 29129140 10.1080/07391102.2017.1404934

[pro70542-bib-0033] Touw WG , Baakman C , Black J , te Beek TAH , Krieger E , Joosten RP , et al. A series of PDB‐related databanks for everyday needs. Nucleic Acids Res. 2015;43:D364–D368. 25352545 10.1093/nar/gku1028 PMC4383885

[pro70542-bib-0034] Vera‐Rodríguez DJ , Sapienza PJ , Popov KI , Lee AL . A flexible, allosteric loop regulates protein activity and rewires electrostatics. Protein Sci. 2025;34:e70315. 40990844 10.1002/pro.70315 PMC12459221

[pro70542-bib-0035] Wang J , Jain A , McDonald LR , Gambogi C , Lee AL , Dokholyan NV . Mapping allosteric communications within individual proteins. Nat Commun. 2020;11:3862. 32737291 10.1038/s41467-020-17618-2 PMC7395124

[pro70542-bib-0036] Watson JL , Juergens D , Bennett NR , Trippe BL , Yim J , Eisenach HE , et al. De novo design of protein structure and function with RFdiffusion. Nature. 2023;620:1089–1100. 37433327 10.1038/s41586-023-06415-8 PMC10468394

[pro70542-bib-0037] Weissenow K , Rost B . Are protein language models the new universal key? Curr Opin Struct Biol. 2025;91:102997. 39921962 10.1016/j.sbi.2025.102997

[pro70542-bib-0038] Wu T , Mu X , Xue Y , Xu Y , Nie Y . Structure‐guided steric hindrance engineering of *Bacillus badius* phenylalanine dehydrogenase for efficient L‐homophenylalanine synthesis. Biotechnol Biofuels. 2021;14:207. 34689801 10.1186/s13068-021-02055-0 PMC8543943

[pro70542-bib-0039] Yang KK , Amini AP . Simplifying protein engineering with deep learning. Cell. 2025;188:4477–4479. 40845808 10.1016/j.cell.2025.07.037

[pro70542-bib-0040] Yu H , Yan Y , Zhang C , Dalby P . Two strategies to engineer flexible loops for improved enzyme thermostability. Sci Rep. 2017;7:41212. 28145457 10.1038/srep41212 PMC5286519

[pro70542-bib-0041] Zeballos N , Ginés‐Alcober I , Macías‐León J , Andrés‐Sanz D , González‐Ramírez AM , Sánchez‐Costa M , et al. Loop engineering of enzymes to control their immobilization and ultimately fabricate more efficient heterogeneous biocatalysts. Protein Sci. 2025;34:e70040. 39840824 10.1002/pro.70040 PMC11751856

[pro70542-bib-0042] Zhu W , Liu Y , Cao H , Liu L , Tan T . Short‐loop engineering strategy for enhancing enzyme thermal stability. iScience. 2025;28:112202. 40212588 10.1016/j.isci.2025.112202 PMC11982487

[pro70542-bib-0043] Zinovjev K , Guénon P , Ramos‐Guzmán CA , Ruiz‐Pernía JJ , Laage D , Tuñón I . Activation and friction in enzymatic loop opening and closing dynamics. Nat Commun. 2024;15:2490. 38509080 10.1038/s41467-024-46723-9 PMC10955111

