# Supplementary material for: SegDesign: A modular framework for controllable protein segment engineering
Source: Protein Sci. 2026 Mar 24;35(4):e70542. doi: 10.1002/pro.70542 (PMC13042651; doi:10.1002/pro.70542)
Supplement: Supplementary file 1 — Table S1. Detailed information for GgTdT design. Table S2. Alignment metrics for fixed regions and redesigned segments in GgTdT variants. Table S3. Representative segment‐level secondary‐structure redesign variants of various enzymes. Table S4. Parameter summary for SegDesign workflow. [file PRO-35-e70542-s001.pdf]

Supplementary Information

SegDesign: a modular framework for controllable protein segment engineering

Chenjie Feng[1,2,3,4], Junbo Yin[1,3,4], Chao Zha[1,3,4], Mohammed Saif[1,3,4], Xiaopeng Xu[1,3,4], Xin Gao[1,3,4,†], Wenjia He [1,3,4,†]

[1]Computer Science Program, Computer, Electrical and Mathematical Sciences and Engineering Division, King Abdullah University of Science and Technology (KAUST), Thuwal 23955-6900, Kingdom of Saudi Arabia.

[2]College of Medical Information and Engineering, Ningxia Medical University, Yinchuan 750004, China;

[3]Center of Excellence for Smart Health (KCSH), King Abdullah University of Science and Technology (KAUST), Thuwal 23955-6900, Kingdom of Saudi Arabia.

[4]Center of Excellence on Generative AI, King Abdullah University of Science and Technology (KAUST), Thuwal 23955-6900, Kingdom of Saudi Arabia.

[†]Corresponding authors:
Xin Gao: xin.gao@kaust.edu.sa
Wenjia He: wenjia.he@kaust.edu.sa

Reproducibility and computational resources

Software, Parameters, and Reproducibility

All software versions, seeds, sampling parameters, and model configurations are logged automatically at each stage. Scripts for executing the SegDesign workflow, along with configuration templates and example datasets, are available in the project repository. All experiments can be reproduced using the provided workflow configuration files and intermediate checkpoints.

Hardware and Runtime

All SegDesign experiments were conducted on NVIDIA A100 GPUs (80 GB VRAM). An backbone generation run producing 100 redesigned backbones typically required ~9 h. For sequence design on a given backbone, ProteinMPNN generated ~1,000 sequences in ~1 h with a batch size of 1.

User Guidelines: Practical recommendations for SegDesign usage

Based on our case studies and empirical experience, we provide the following practical guidelines to facilitate effective use of SegDesign in different design scenarios:

1. **Backbone sampling strategy.**
   We recommend starting with a small number of backbone samples (e.g., ~10 backbones) as a pilot run to rapidly assess whether a given segment is amenable to α-helix or β-strand conversion. Once feasibility is established, sampling can be scaled up. As a general rule, ~100 backbones per objective are sufficient for helix induction, whereas β-strand targets typically require increased sampling (approximately 200–500 backbones) due to stronger contextual constraints.SS3 compliance threshold: use ⩾20-40% as a default, but relax for short strands and tighten for helices.
2. **SS3 compliance threshold.**
   A default SS3 compliance threshold of ~20–40% is generally effective. This threshold can be relaxed for short or context-limited β-strands and tightened for α-helices, which are typically easier to induce and stabilize.
3. **Sequence sampling depth.**
   Sampling 100–1,000 sequences per backbone using ProteinMPNN provides a reasonable balance between sequence diversity and computational cost in most applications. The sampling temperature is typically set to 0.3. Lower values (e.g., 0.1) can be used to generate more conservative sequences, whereas higher temperatures promote increased sequence diversity.
4. **Inverse-folding score filtering.**
   Strict filtering based on ProteinMPNN global scores is usually unnecessary. In practice, accepting sequences with global scores below or near the median is sufficient, and the threshold can be relaxed further depending on downstream validation requirements.
5. **Recommended filtering order.**
   We suggest applying filters in the following order to efficiently reduce the candidate pool while preserving promising designs:
   (i) Backbone SS3 compliance →
   (ii) Backbone SS3 coverage →
   (iii) Inverse-folding score →
   (iv) ESMFold SS3 compliance →
   (v) ESMFold confidence metrics →
   (vi) AlphaFold3 evaluation for representative candidates
6. **Challenges in β-strand design.**
   When β-strand induction proves difficult, extending the design boundaries by 1–3 residues can provide additional sheet context and improve success rates. Very short loops (e.g., 3–5 residues) often exhibit extremely low conversion success, likely because their local structural environment lacks sufficient physical constraints or flexibility to stabilize α-helical or β-strand conformations.
7. **Random seed exploration.**
   Using multiple random seeds during backbone generation can increase structural diversity, particularly for helices, which may be realized through multiple geometrically distinct backbone conformations.
8. **Iterative design strategy.**
   If a single design round yields limited SS3 coverage (e.g., ~40% helix compliance), iterative refinement can be effective. In such cases, partially successful designs can be used as input scaffolds for subsequent SegDesign rounds to progressively enrich the desired secondary-structure features.

Supplementary Tables

Table S1. Detailed information for *Gg*TdT design.

Strategy	#Backbones passing structural filters	#Sequences passing inverse folding	MMseqs2 min_seq_id	#Representative variants after clustering	#Structurally validated variants	Mean segment pLDDT[*]	Mean segment SS3 compliance rate[*]
**L1H**	18	9012	0.96	647	526	54.6	58%
**L2H**	33	24000	0.94	71	44	55.3	50%
**L2S**	5	1507	0.95	556	33	66.5	52%

[*]Mean segment pLDDT is calculated among structurally validated variants.

Table S2. Alignment metrics for fixed regions and redesigned segments in GgTdT variants.

Strategy	Variant	Region	Fixed region RMSD (Å)[*]	Fixed region TM-score[*]	Designed segment RMSD (Å)[*]	Designed segment TM-score[*]
**L1H**	rfd_40_mpnn_399	230-245	2.92	0.92	3.26	0.08
	rfd_6_mpnn_862	230-245	2.67	0.92	4.08	0.03
**L2H**	rfd_54_mpnn_440	281-305	3.01	0.88	15.72	0.03
	rfd_69_mpnn_500	281-305	3.20	0.89	14.61	0.02
**L2S**	rfd_40_mpnn_381	281-305	3.55	0.94	15.75	0.10
	rfd_86_mpnn_508	281-305	2.98	0.91	12.42	0.06

[*]Cα RMSD and TM-score were computed using Cα atoms after global rigid-body superposition based on one-to-one residue correspondence between wild-type and designed structures.

Table S3. Representative segment-level secondary-structure redesign variants of various enzymes

Enzyme	Target secondary structure	Segment	DSSP SS3 (before design)	Segment SS3 (after design)	Segment H%	Segment E%	Segment C%
**Adenylate kinase**	α-helix	211-217	CHHHCCC	CC**HHHH**C	**0.57**	0.00	0.43
**Haloalkane dehalogenase**	β-strand	1-12	CCCCCCCCCCCC	C**EEEE**CC**EEE**CC	0.00	**0.58**	0.42
**Beta-lactamase TEM**	α-helix	211-225	CCCCCCCCHHHHCCC	C**HHH**CC**HHHHHH**CCC	**0.60**	0.00	0.40
**Klebsiella pneumoniae D-lactate dehydrogenase (LDHD)**	α-helix	129-145	CCCCCCCCCCCCC<br>CCCCCCCCCCCCC	CCCCCC**HHHHHHHH**<br>**HHHHH**CCC**HHHH**C	**0.62**	0.00	0.38
**Klebsiella pneumoniae D-lactate dehydrogenase (LDHD)**	β-strand	129-145	CCCCCCCCCCCCC<br>CCCCCCCCCCCCC	CCCCCCC**EEEEE**C<br>C**EEEE**CHHHHHCC	0.19	**0.35**	0.46
**Aspergillus flavus urate oxidase (UOX)**	β-strand	104-128	CCCCEEEEECCEE<br>EEEEEECCCCCC	CCC**EEEEEEE**CC**EE**<br>**EEEEEEE**CCCCC	0.00	**0.60**	0.40

Table S4. Parameter summary for SegDesign workflow

Module	Parameter	Meaning / Role	Example / Default	Notes (Filter/Threshold)
**HMMER (Module 1)**	bitscore	HMMER conservation bit-score threshold	0.3	controls region selection for evolutionary conservation
	n_iter	Number of JackHMMER iterations	5	more iterations → deeper alignment
	cpu	Number of CPU cores	10	parallel efficiency
	threshold	Conservation threshold	0.6	used for identifying conservative segments
**RFdiffusion (Module 2)**	num_designs	Backbone designs per segment	10	number of backbone samples
	threshold	Design quality threshold	0.4	filter on design scoring (interpreted post-generation)
	helix	Helix constraint flag	false	enable helix SS3 target
	strand	Strand constraint flag	false	enable strand SS3 target
**ProteinMPNN (Module 3)**	num_seq_per_target	Sequences sampled per backbone	20	number of sampled sequences per backbone
	sampling_temp	MPNN sampling temperature	0.3	controls the diversity of designed sequences
	seed	Random seed	42	reproducibility
	top_percent	Top selection percentile	0.9	filter for best sequences
**ESMFold validation (Module 4)**	ptm_threshold	Predicted TM (pTM) threshold	0.54	structural plausibility filter
	plddt_threshold	pLDDT confidence threshold	70	removes low-confidence designs
